# Evidence for a Functional HipBA Toxin–Antitoxin System in *Acidovorax citrulli*

**DOI:** 10.3390/ijms26073366

**Published:** 2025-04-03

**Authors:** Hao Zhang, Mei Zhao, Lulu Cai, Wei Guan, Yuwen Yang, Ron Walcott, Wenjun Zhao, Tingchang Zhao

**Affiliations:** 1 College of Plant Protection, Jilin Agricultural University, Changchun 130118, China; hao980410@163.com; 2 Institute of Plant Protection, Chinese Academy of Agricultural Sciences, Beijing 100193, China; ywyang@ippcaas.cn; 3 Department of Plant Pathology, College of Plant Protection, China Agricultural University, Beijing 100193, China; 4 Center for Biosafety, Chinese Academy of Inspection and Quarantine, Sanya 572024, China; cailulu20062@163.com (L.C.); wenjunzhao@188.com (W.Z.); 5 Department of Plant Pathology, University of Georgia, Athens, GA 30602, USA; rwalcott@uga.edu

**Keywords:** *Acidovorax citrulli*, HipBA, toxin–antitoxin systems, stress response

## Abstract

Bacterial fruit blotch (BFB) is a highly destructive seed-borne and seed-transmitted disease caused by the Gram-negative bacterium *Acidovorax citrulli* that has caused substantial economic losses for the cucurbit industry in China. Despite its potential for economic damage, little is known about the bacterium’s molecular mechanisms of pathogenicity. Toxin–antitoxin (TA) systems are critical for the bacterial stress response. These systems are composed of two genes, toxin and antitoxin, that encode a stable toxin protein and a labile antitoxin protein, respectively. In this study, the genes for the putative HipBA TA system were identified in *A. citrulli* genomes through bioinformatic analysis. A series of molecular biology experiments have demonstrated that the HipBA TA system exists in *A. citrulli* Aac5. Furthermore, the transcription of *hipA* and *hipB* in *A. citrulli* Aac5 were induced by pH stress, chloramphenicol stress, and during plant infection. Overall, our results have revealed an active type II TA system, HipBA, in *A. citrulli* Aac5, and provided insights into its biological functions. These findings contribute to a better understanding of TA systems in plant pathogens.

## 1. Introduction

Bacterial fruit blotch (BFB) is a seed-borne and seed-transmitted disease that can cause severe damage to cucurbit crops, such as watermelon and melon [1]. It is prevalent in several provinces in China, especially Xinjiang and Hainan, and has also been reported in other countries such as the United States [2], Australia [3], Hungary [4], and Brazil [5], negatively impacting the productivity of the cucurbit industry. The disease is caused by the Gram-negative bacterium *Acidovorax citrulli* [6]. An important primary source of inoculum for BFB is cucurbit seeds infected with *A. citrulli*. The initial BFB symptoms may include water-soaked spots on cotyledons and leaves of cucurbit seedlings and plants that eventually develop into brown lesions. Infected watermelon and melon fruit can develop water-soaked, irregularly shaped, sunken lesions and rot [1,7]. The seeds inside infected fruits may become contaminated with *A. citrulli* [8].

The current methods for BFB management include traditional pathogen exclusion by quarantine and seed health testing and pathogen eradication by chemical and biological control treatments of seeds [9]. Despite this, a clear understanding of the pathogenic mechanisms employed by *A. citrulli* is still lacking, and there is a need to improve BFB management strategies through the development of host plant resistance to more effectively control BFB [10,11].

Toxin–antitoxin (TA) systems are self-regulating systems widely present in the chromosomes and plasmids of environmental and pathogenic microorganisms [12,13,14]. These systems consist of two adjacent genes, one of which encodes a toxin that inhibits bacterial growth and the other encodes an antitoxin that neutralizes the toxin’s effects [15,16]. TA systems are classified into eight types based on the biological properties of the antitoxins and how they neutralize their corresponding toxins [17]. Type II TA systems have been the most extensively studied among the eight types. In type II TA systems, the toxin and antitoxin genes are usually located on the same operon and are co-transcribed. Antitoxin proteins form stable complexes with toxin proteins to neutralize their toxic effects. The antitoxin proteins can bind to their own promoter to negatively regulate their own transcription and have a stronger autoregulatory effect when bound to the promoter as a toxin–antitoxin complex [18]. Under specific, mostly stressful conditions, the unstable antitoxin is degraded, releasing the toxin and leading to cell growth arrest [19].

Research findings have suggested that TA systems play a role in stress response and antibiotic resistance [20]. For instance, treatment with the antibiotic chloramphenicol significantly increased the transcription levels of type II TA system genes *vapB* and *vapC* in *A. citrulli* 7a1. Furthermore, *vapB* and *vapC* have been implicated in plant pathogenesis [21]. In the type II TA system HipBA of *Escherichia coli*, the interaction between the toxin protein HipA and the antitoxin protein HipB neutralizes the toxic effect of the former. Additionally, the association between HipA in the TA system and bacterial antibiotic resistance has been reported [22].

HipBA is a common type II TA system in pathogenic bacteria [23]. It is composed of two genes, *hipA* and *hipB*. *hipA* encodes a toxin that inhibits cell growth, while *hipB* encodes an antitoxin that neutralizes the HipA’s toxic effects [24]. These proteins form a complex that binds to the promoter region of the *hipBA* operon through the DNA-binding domain of HipB, negatively regulating the transcription of the *hipBA* operon [25]. Under adverse conditions, the less-ordered structure of the antitoxin protein, HipB, makes it unstable and susceptible to degradation by specific proteases. This results in the release of the toxin protein HipA, which exerts its toxic effects and induces dormancy in the cells [26].

HipBA is not only involved in bacterial cell growth, but also antibiotic resistance [27]. Although TA systems have been extensively studied in human and animal pathogenic bacteria, research on these systems in plant pathogenic bacteria is limited. In this study, we identified HipBA as a type II TA system in *A. citrulli* Aac5 and found that *hipA* and *hipB* were involved in the pathogen’s response to pH and chloramphenicol stress and played a role in plant pathogenesis.

## 2. Results

### 2.1. Bioinformatic Analysis of hipA and hipB in Acidovorax citrulli

**Figure 1:**
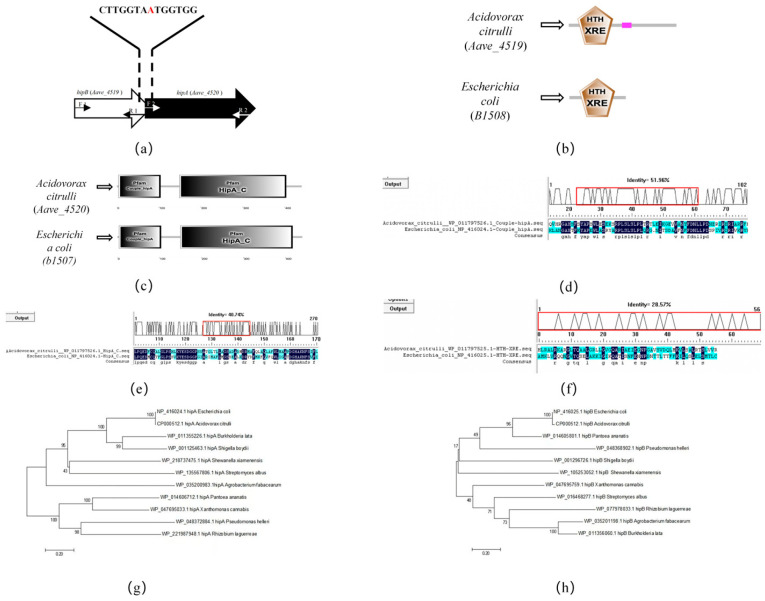
Bioinformatic analysis of HipA and HipB in *Acidovorax citrulli*. (**a**) Diagram illustrating the position of *hipA* and *hipB* genes in the *A. citrulli* AAC00-1 genome (Genbank: CP000512.1), with the overlapping region highlighted in red. Arrows indicate primer positions for the electrophoresis analysis presented in Figure 2. (**b**) Domain analysis of HipA using the SMART tool (http://version10.string-db.org/, accessed on 13 April 2021). (**c**) Domain analysis of HipB using the SMART tool. (**d**) Amino acid sequence comparison of *A. citrulli* Aac5 and *Escherichia coli* Couple_hipA domains using DNAMAN, showing a similarity of 51.96%. The red box indicates the currently selected visible region in the alignment results. (**e**) Amino acid sequence comparison of *A. citrulli* Aac5 and *E. coli* HipA_C domains using DNAMAN, showing a similarity of 40.74%. The red box indicates the currently selected visible region in the alignment results. (**f**) Amino acid sequence comparison of *A. citrulli* Aac5 and *E. coli* HipB domains using DNAMAN, showing a similarity of 28.57%. The red box indicates the currently selected visible region in the alignment results. (**g**) Phylogenetic tree of the *hipA* gene. (**h**) Phylogenetic tree of the *hipB* gene.

The bioinformatics analysis of the genomes of *A. citrulli* strains AAC00-1, pslb65, and DSM17060 revealed the presence of the *hipA* and *hipB* genes. In strain AAC00-1, the genes *Aave_4520* (*hipA*) and *Aave_4519* (*hipB*) showed a high degree of homology with *E. coli*’s *hipA* and *hipB* genes. In the genome of AAC00-1, these genes overlapped by one bp and had the same transcription direction (Figure 1a), suggesting that they functioned as an operon. Further analysis of *A. citrulli* strain Aac5 showed that the HipA and HipB proteins shared the same domains as their counterparts in *E. coli*. HipB in Aac5 contains a DNA-binding HTH_XRE domain (Figure 1b), commonly found in transcriptional regulators. In Aac5 HipA, the Couple_hipA and HipA_C domains were identified (Figure 1c). The Couple_hipA domain is a bacterial serine/threonine protein kinase involved in multidrug tolerance [28]. The HipA_C domain is known to be involved in high-frequency persistence to the lethal effects of inhibiting DNA or peptidoglycan synthesis [29]. When expressed alone, HipA can be toxic to bacterial cells, but it is typically tightly associated with HipB, and the HipBA complex may be involved in autoregulation of the *hipBA* operon [30]. A comparison of the amino acid sequences of HipA and HipB domains in *E. coli* and *A. citrulli* revealed similarities between the Couple_hipA domain in *A. citrulli* and *E. coli* (51.96%) (Figure 1d), HipA_C in *A. citrulli* and *E. coli* (40.74%) (Figure 1e), and HTH_XRE in *A. citrulli* and *E. coli* (28.57%) (Figure 1f). Phylogenetic analysis based on *hipA* (Figure 1g) and *hipB* (Figure 1h) showed that *hipA* and *hipB* in *E. coli* were closely related to the same genes in *A. citrulli*. These findings indicate that the proteins encoded by *hipA* and *hipB* in *A. citrulli* Aac5 are similar to those of *E. coli* HipA and HipB, suggesting the presence of a type II TA system in *A. citrulli*.

### 2.2. Co-Transcription Test

**Figure 2:**
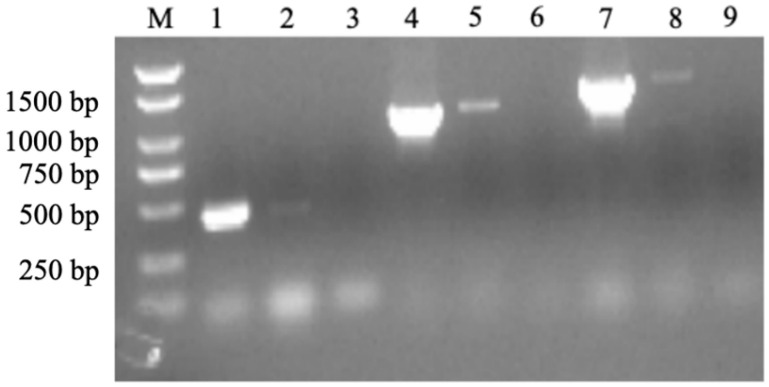
PCR amplification results obtained using cDNA products and different primer sets. Lanes 1, 4, and 7 represent the amplification products using cDNA as the template with *hipB* internal primers (*hipB* F 1 and *hipB* R 1; Table A2), *hipA* internal primers (*hipA* F 2 and *hipA* R 2; Table A2), and primers spanning *hipA* and *hipB* open reading frames (*hipB* F 1 and *hipA* R 2), respectively. Lanes 2, 5, and 8 represent PCR-positive controls using the genomic DNA of strain *A. citrulli* Aac5 as templates, corresponding to the same order of primers as in Lanes 1, 4, and 7. Lanes 3, 6, and 9 represent PCR products using ultra-pure water as templates, corresponding to the same order of primers as those in Lanes 1, 4, and 7. M: Trans 2K Plus DNA Marker.

In the conventional type II TA system, the toxin and antitoxin genes are co-transcribed [31,32]. To investigate the co-transcription of the *hipA* and *hipB* in *A. citrulli* Aac5, the transcriptional activities of these genes were examined. As shown in Figure 2, the PCR products obtained using DNA and cDNA templates were of the same size, indicating that the *hipA* toxin gene and the *hipB* antitoxin gene are co-transcribed. This finding suggests that *hipA* and *hipB* may comprise a TA system in *A. citrulli*.

### 2.3. HipB Binds to the Promoter of the hipBA Operon Region in Aac5

**Figure 3:**
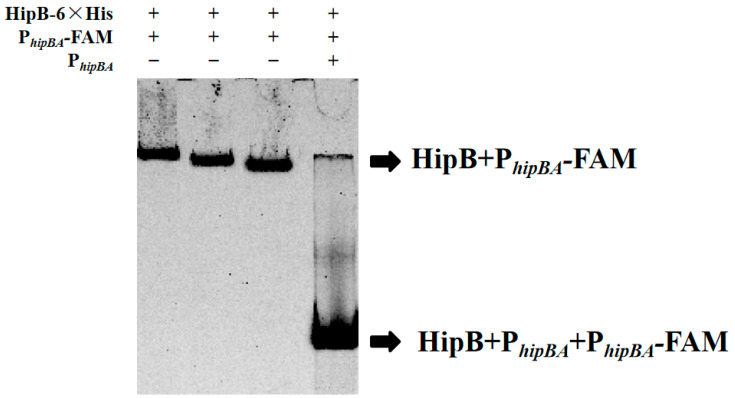
The electrophoretic mobility shift assay (EMSA) of HipB with its own promoter. Direct interaction between the HipB protein and the *hipBA* promoter DNA labeled with 6-carboxy-fluorescein (FAM) was detected by EMSA. Shifted bands indicate the binding of the probe DNA (P*_hipBA_*-FAM) to the HipB protein. P*_hipBA_* represents the promoter sequence of *hipBA*. The P*_hipBA_* was added to the incubation system to compete with the interaction between HipB and the FAM-labeled probe. P*_hipBA_*-FAM represents the fluorophore FAM attached to the 5′ of P*_hipBA_*.

In a typical type II TA system, the antitoxin protein regulates its own transcription and that of the toxin protein by binding to its own promoter via a DNA-binding domain [33]. To investigate whether the putative HipB protein in *A. citrulli* Aac5 can bind to the promoter of the *hipBA* operon region, an electrophoretic mobility shift assay (EMSA) was conducted. The EMSA results demonstrated that the HipB protein specifically binds to the promoter of the *hipBA* operon. As summarized in Table A3 and shown in Figure 3, a clear concentration-dependent shift was observed in the first three lanes (Systems 1–3), where reducing the amounts of the HipB protein led to progressively stronger band shifts. This indicated the formation of a HipB–DNA complex. Specifically, Lane 1, with the highest HipB concentration, exhibited the slowest migration (most pronounced shift), while reduced protein levels in Lanes 2 and 3 resulted in progressively faster migration (weaker shifts).

Furthermore, a competition assay (Lane 4) using unlabeled P*_hipBA_* as a competitor significantly reduced the binding of HipB to the labeled probe, resulting in the fastest migration (weakest shift). This was evidenced by the absence of a shifted band. These observations confirm the specific and competitive nature of HipB binding to the *hipBA* operon promoter region and demonstrate that HipB binds to the promoter in a specific and concentration-dependent manner.

### 2.4. Effect of Heterologous Expression of Putative HipA and HipB on E. coli Growth

To investigate the biological functions of the candidate proteins HipA and HipB in *A. citrulli*, the heterologous expression in *E. coli* BL21 [23] was employed (*E. coli* BL21 was selected due to its high efficiency in protein expression and lack of endogenous proteases, such as Lon and OmpT, which reduce the degradation of expressed proteins). This approach was chosen to independently validate the functions of the toxin gene (*hipA*) and antitoxin gene (*hipB*), as the native genetic background of *A. citrulli* could interfere with the interpretation of results. Direct knockout experiments in *A. citrulli* were unsuccessful, as the deletion of either gene led to bacterial death, suggesting that these genes are essential for survival. Furthermore, the overexpression of *hipA* in *A. citrulli* was lethal, further highlighting the toxic nature of HipA. These challenges in the native host underscore the necessity of using *E. coli* as a simplified system to study the functions of these genes.

**Figure 4:**
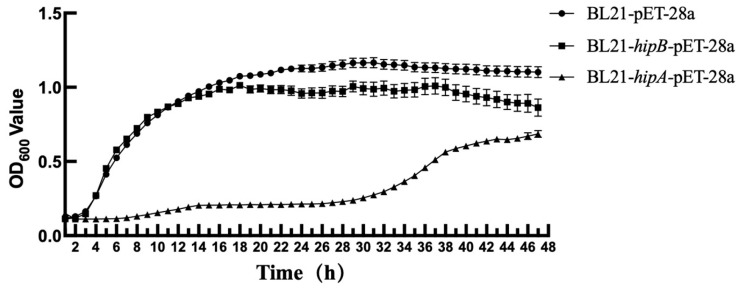
Growth curve of *Escherichia coli* BL21 transformed with pET-28a plasmids expressing recombinant *hipA* or *hipB* from *A. citrulli* separately. The control group was BL21 with an empty vector pET-28a, while BL21-*hipA* and BL21-*hipB* were plasmids containing *hipA* and *hipB* genes from *A. citrulli*, respectively, that were transformed into *E. coli* BL21. The OD_600_ value was measured every 2 h for 72 h (Bioscreen C PRO, Finland). Each treatment included ten replicates, and the experiment was independently conducted three times.

The population growth of *E. coli* cells expressing HipB from *A. citrulli* was comparable to that of wild-type *E. coli* (BL21-pET-28a) during the initial 12 h of incubation, indicating that HipB expression alone does not affect *E. coli* growth (Figure 4). However, when HipA from *A. citrulli* alone was induced in *E. coli*, the growth was significantly reduced compared to that of the wild-type *E. coli* (BL21-pET-28a) for the first 32 h. This suggests that expressing HipA from *A. citrulli* has a considerable inhibitory effect on *E. coli* growth.

### 2.5. Bacterial Two-Hybrid Experiment

**Figure 5:**
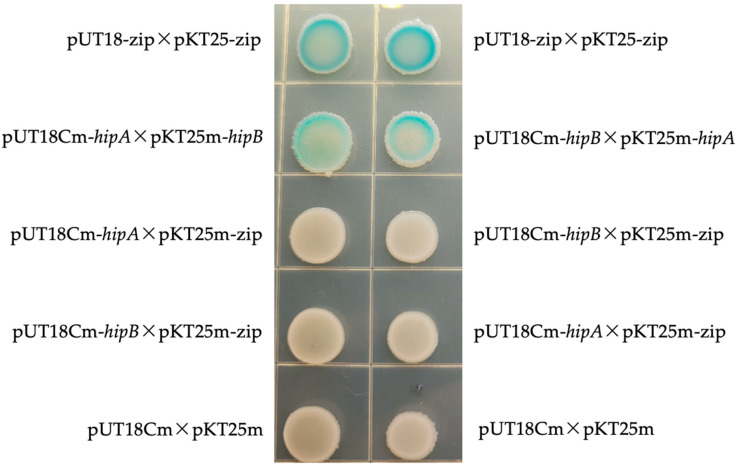
A bacterial two-hybrid experiment was used to demonstrate the interaction between HipA and HipB. pKT25-zip × pUT18-zip was used as a positive control; and pUT18Cm-*hipA* × pKT25m-zip, pUT18Cm-*hipB* × pKT25m-zip, pUT18Cm-*hipA* × pKT25m-zip, and pKT25m × pUT18Cm were negative controls. The experimental group consisted of pUT18Cm-*hipA* × pKT25m-*hipB* and pUT18Cm-*hipB* × pKT25m-*hipA*. The experiment was performed three times, with consistent results each time.

To confirm interaction between HipA and HipB proteins in *A. citrulli* Aac5, both yeast two-hybrid and bacterial two-hybrid experiments were conducted. In the yeast two-hybrid experiment, the target genes were fused with pGADT7 and pGBKT7 vectors and co-transformed into *Saccharomyces cerevisiae* Y2HGold. Although the yeast two-hybrid assay did not yield positive results on selective media (SD/-Leu/-Trp/-His/-Ade), potentially due to the limitations of the yeast system or environmental conditions, the bacterial two-hybrid experiment successfully confirmed the interaction between HipA and HipB. In the bacterial system, the target gene was amplified through PCR, fused with the plasmids pKT25m and pUT18Cm, and the resulting recombinant vectors were co-transformed into *E. coli* BTH101. The experimental group (pUT18Cm-*hipA* × pKT25m-*hipB*; pUT18Cm-*hipB* × pKT25m-*hipA*) and the positive control group (pUT18-zip × pKT25-zip) showed blue colonies on the LB + Amp + Kan agar (Figure 5). In contrast, the negative control group (pUT18Cm-*hipA* × pKT25m-*zip*; pUT18Cm-*hipB* × pKT25m-*zip*; pUT18Cm-*hipB* × pKT25m-*zip*; pUT18Cm-*hipA* × pKT25m-*zip*; pUT18Cm × pKT25m) did not turn blue on the LB + Amp + Kan solid medium. These results confirm the interaction between HipA and HipB proteins in *A. citrulli* Aac5.

### 2.6. Quantitative Real-Time PCR Analyses of hipA and hipB mRNA Levels in A. citrulli Aac5 Under Various Environmental Stress Conditions

#### 2.6.1. Analysis of *hipA* and *hipB* Transcription Levels in Aac5 Under Chloramphenicol Stress

**Figure 6:**
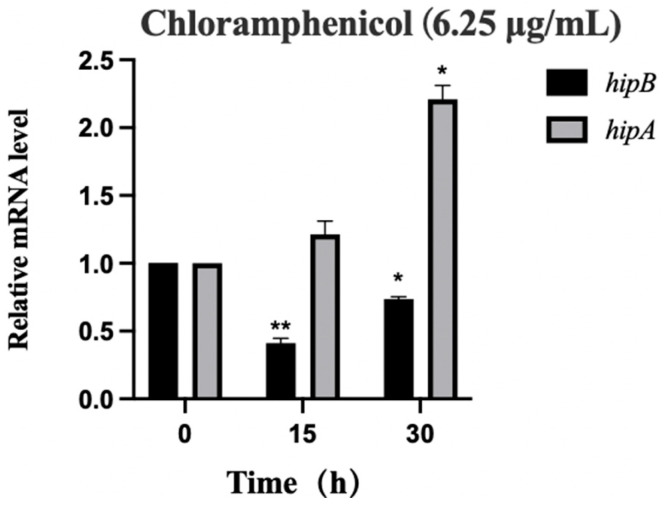
Transcriptional activation of *hipA* and *hipB* in *Acidovorax citrulli* Aac5 under chloramphenicol stress (6.25 μg/mL). The expression levels of each gene at 0 h in wild-type strain Aac5 were used as the reference and normalized to 1. Data analysis was performed using the 2^−ΔΔCt^ method. Statistical analysis was conducted using Prism 9 software, (*: *p* < 0.05; **: *p* < 0.01). The experiment was conducted three times, and consistent results were obtained each time.

To induce stress in Aac5, a concentration of 6.25 μg/mL of chloramphenicol was selected based on the preliminary observations. We selected 15 h and 30 h as observation points to investigate the long-term adaptation of *A. citrulli* to chloramphenicol stress, considering that TA systems may regulate persister cell formation over extended periods. Previous studies have indicated that prolonged antibiotic exposure can induce TA-mediated dormancy [24]. Our results showed that chloramphenicol exposure (6.25 μg/mL) had a significant impact (*p* ≤ 0.05) on the transcription levels of *hipA* and *hipB* in Aac5 (Figure 6). After 15 h of chloramphenicol exposure, there was a significant decrease (*p* ≤ 0.01) in the *hipB* mRNA levels, while, after 30 h, the *hipA* mRNA levels increased by 1.2 compared to that of the initial measurement, and the *hipB* levels decreased by 0.3. This trend suggests a possible transition to a persister-like state, where increased toxin activity aids bacterial survival by reducing metabolic activity, while prolonged stress conditions may lead to global transcriptional regulation changes affecting *hipB* stability. These results have demonstrated that Aac5 activated the expression of *hipA* and *hipB* under chloramphenicol stress.

#### 2.6.2. Transcription Level Analysis of *hipA* and *hipB* in Aac5 Under pH Stress

**Figure 7:**
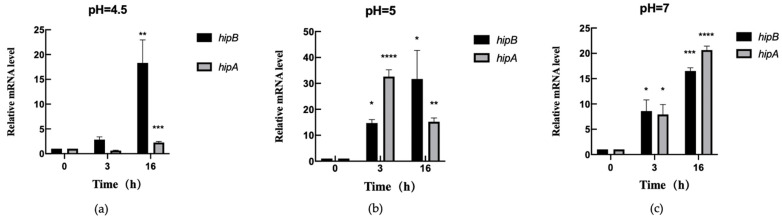
Transcription levels of *hipA* and *hipB* under pH stress in Aac5. The transcription levels of *hipA* and *hipB* were measured using quantitative real-time (qRT) PCR under environmental stress of pH 4.5 (**a**), pH 5 (**b**), and pH 7 (**c**). The expression levels of each gene in the wild-type strain Aac5 at 0 h were set as 1. The data were analyzed via 2^−ΔΔCt^. Statistical analysis was performed using Prism 9 software, where * indicates *p* < 0.05; ** indicates *p* < 0.01; *** indicates *p* < 0.001; and **** indicates *p* < 0.0001. The experiment was conducted three times, and consistent results were obtained each time.

To investigate the growth ability of Aac5 under different concentrations of acid–alkali stress, a transcription level analysis was conducted at pH values of 4.5, 5, and 7. The results showed that, after 3 h of exposure to pH 4.5, the expression level of *hipA* remained unchanged compared to that observed at 0 h, while the *hipB* expression levels were slightly up-regulated but not statistically significant (Figure 7a). However, after 16 h, both *hipA* and *hipB* expression levels were up-regulated, with *hipB* showing significantly higher expression than *hipA* (Figure 7a). These findings suggest that Aac5 activated the induced expression of both *hipA* and *hipB* under acidic conditions.

At pH 5, compared to those observed at 0 h, the gene expression levels of *hipA* and *hipB* were significantly up-regulated in Aac5 at 3 h. The expression level of *hipA* was higher than that of *hipB* (Figure 7b). After 16 h, the transcription levels of both *hipA* and *hipB* significantly increased compared to those observed at 0 h, with *hipB* showing a higher transcription level than *hipA*.

Similarly, at pH 7, the expression levels of *hipA* and *hipB* were significantly up-regulated at 3 h compared to those observed at 0 h, with *hipB* showing a slightly higher transcription level than *hipA* (Figure 7c). After 16 h, the transcription levels of both *hipA* and *hipB* significantly increased compared to those observed at 0 h, with *hipA* showing a higher transcription level than *hipB*.

#### 2.6.3. Differential Expression of *hipA* and *hipB* in Aac5 During Pathogen–Host Interaction

**Figure 8:**
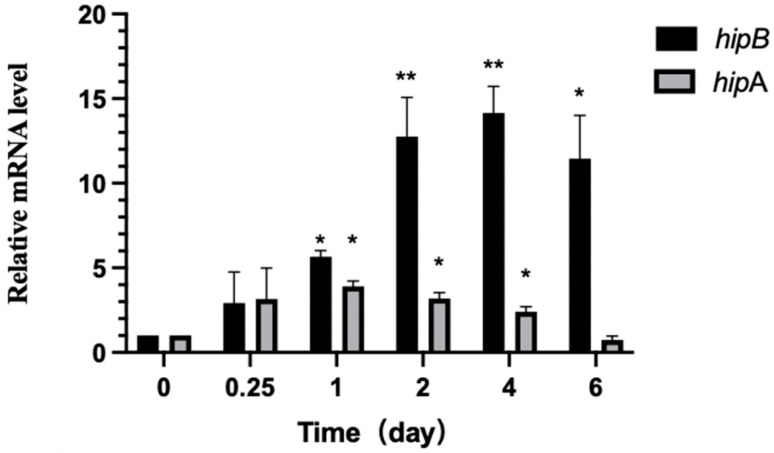
Transcription levels of *hipA* and *hipB* during pathogen–host interaction in Aac5. The expression levels of each gene at 0 h in wild-type strain Aac5 were normalized to 1, and the data were analyzed using the 2^−ΔΔCt^ method. Statistical analysis was performed using Prism 9 software, with * indicating *p* < 0.05 and ** indicating *p* < 0.01. The experiment was conducted three times, and similar results were obtained each time.

To investigate the role of the TA system in regulating pathogen growth during host infection, we monitored the transcription levels of *A. citrulli hipBA* at various time points after inoculating watermelon cotyledons. Our results indicate that *hipA* and *hipB* of *A. citrulli* were activated during the infection process (Figure 8). Specifically, *hipA* transcription levels significantly increased (*p* ≤ 0.05) at 1 day after inoculation (dai) compared to those observed at 0 h, while *hipB* exhibited higher expression relative to that observed at 0 h. Over longer time periods, the *hipB* transcription levels continued to increase more than *hipA*. At 2 and 4 dai, the *hipA* transcription levels remained significantly higher (*p* ≤ 0.05) than those observed at 0 h, with minimal change, whereas *hipB* expression continued to rise during the same period. A decreasing trend in *hipA* expression over time was observed, and, by 6 dai, its expression was not significantly different from that observed at 0 h. However, the *hipB* mRNA levels were still significantly (*p* ≤ 0.05) higher at 4 and 6 dai compared to those observed at 0 h. Notably, the expression of the antitoxin gene *hipB* was significantly higher than that of the toxin gene *hipA* during the 1–6 dai. This may be due to the instability of the antitoxin, which requires a high concentration to neutralize the toxin and prevent the release of toxin HipA.

## 3. Discussion

This study aimed to identify and characterize a toxin–antitoxin system in *A. citrulli* Aac5. The type II TA system has gained attention in recent years because of its association with bacterial stress resistance [20,21]. BFB has the potential to negatively impact watermelon and melon production globally. However, the molecular mechanisms of *A. citrulli* virulence have not yet been fully elucidated. Through sequence analysis, a putative TA system was discovered in the genome of *A. citrulli* AAC00-1, which showed homology to the validated type II TA system HipBA found in other bacterial species. This study found that *hipA* (*Aave_4520*) and *hipB* (*Aave_4519*) were adjacent to each other in the genome of *A. citrulli* AAC00-1, with *hipA* being located downstream of *hipB*, and overlapping by one base. This is a typical feature of most type II TA systems. Previous studies have shown that the toxin protein HipA typically inhibits DNA replication or protein translation, resulting in cell growth stagnation [34]. In this study, the heterologous expression of the candidate protein HipA from *A. citrulli* Aac5 in *E. coli* led to significantly delayed growth, consistent with the growth retardation effect of HipA on host cells. In most type II TA systems, the combination of antitoxins and toxins can counteract the toxin activity. In this study, the protein interaction between the candidate proteins HipA and HipB was verified using the bacterial two-hybrid method, indicating an interaction between HipA and HipB in Aac5. In the traditional type II TA systems, the antitoxin protein binds to its own promoter to regulate transcription [35]. Therefore, the EMSA experiment showed that candidate protein HipB can bind to the promoter of its own operon and preliminarily verified that candidate HipB from *A. citrulli* exhibits characteristics of an antitoxin protein. The above results confirmed that HipBA is a TA system in *A. citrulli* Aac5.

In this study, we showed that the transcription of *hipA* and *hipB* is induced under pH and chloramphenicol stress. Previous research has shown that the levels of toxin and antitoxin in bacteria are low under normal growth conditions due to self-regulation of the type II TA system [36]. Our results showed that the expression levels of *hipA* and *hipB* genes changed in response to pH and chloramphenicol stress. Moreover, relevant research showed that, under external pressure, antitoxin is degraded, weakening or relieving the self-regulation effect and leading to changes in the transcription of the *hipA* and *hipB* genes in the TA system [32]. Our experimental data corroborate that pH 7 is the optimal pH for *A. citrulli* growth, as validated through growth curve analyses. Using this as a neutral reaction condition, we observed that both the toxin (*hipA*) and antitoxin (*hipB*) genes were actively transcribed and expressed, with expression gradually increasing over time. The simultaneous increase in *hipA* and *hipB* under neutral conditions suggests a balanced regulation of the TA system, potentially maintaining a basal level of toxin and antitoxin to ensure proper cellular function and preparedness for potential stress. Moreover, we observed that, at pH 4.5, *hipB* expression surpasses *hipA* after 16 h, whereas, at pH 5, *hipA* initially dominates. This inconsistency can be attributed to several mechanisms, including differential protease activity or promoter regulation under varying pH conditions. For instance, at pH 4.5, stronger acid stress may activate additional stress-response pathways, leading to enhanced *hipB* expression over time as a protective mechanism. Conversely, at pH 5, the milder acid stress may allow for a different regulatory pattern where *hipA* dominance primes cells for potential growth arrest. This suggests that, under adverse conditions, Aac5 participates in self-regulation through *hipA* and *hipB*. Unlike typical short-term TA system activation, our results suggest that HipBA may be involved in long-term bacterial survival strategies. The prolonged upregulation of *hipA* at 30 h, coupled with *hipB* downregulation, indicates a shift towards a persister state rather than an immediate toxin–antitoxin equilibrium shift. This aligns with previous studies showing that TA systems play a role in persister cell formation under antibiotic stress [24].

Our study demonstrates that HipBA is involved in *A. citrulli* pathogenesis. Recent research has shown that the TA system is implicated in this interaction. For example, Bodogai et al. found that NtrPR, a VapBC-like module of *Sinorhizobium meliloti*, helps to regulate metabolic levels during beneficial symbiosis with plants, with increased mRNA levels of *vapB* and *vapC* after inoculation [37]. Shavit et al. reported that the *A. citrulli* VapBC system was involved in plant pathogenesis [22]. Our experiments have further demonstrated that the expression of *hipA* and *hipB* in *A. citrulli* Aac5 was induced during pathogen infection, indicating that HipBA plays a regulatory role in the interaction between the pathogen and the plant host.

Previous studies have demonstrated that TA systems can influence persistent bacterial infection by regulating metabolic activities. For example, the NtrPR (VapBC-like TA system) in Sinorhizobium meliloti has been shown to modulate metabolic levels during plant symbiosis, enabling bacterial adaptation to the host environment [38]. Similarly, the VapBC system in *A. citrulli* has been reported to be involved in plant pathogenesis [37,39]. In our study, *hipA* and *hipB* expression was induced during host infection in Aac5, with *hipB* consistently being expressed at a higher level than *hipA*. This pattern aligns with the role of TA systems in bacterial persistence and metabolic regulation. Moreover, TA systems have been shown to contribute to biofilm formation. Some TA systems promote persister cell formation by inhibiting cell growth and altering metabolic states, thereby enhancing bacterial adaptation to the host environment [40]. Based on our experimental data, HipBA may influence the persistence of *A. citrulli* within the host, indirectly affecting biofilm formation rather than directly regulating the expression of virulence factors. This unique mechanism suggests that HipBA plays a distinctive role in the pathogenesis of *A. citrulli* and may serve as a potential target for controlling bacterial fruit blotch (BFB).

## 4. Materials and Methods

### 4.1. Bacterial Strains, Plasmids, Plant Materials, and Growth Conditions

The bacterial strains and plasmids used in this study are listed in Table A1. The watermelon cultivar ‘Ruixin’ was purchased from China Vegetable Seed Technology Corporation, Beijing, China. *Acidovorax citrulli* Aac5 was grown in King’s B medium [28] at 28 °C, with constant shaking at 225 rpm. *Escherichia coli* strains BL21 and BTH101 were routinely grown in Lysogeny broth (LB) [28] at 37 °C, with constant shaking at 225 rpm. All strains were stored as glycerol stocks at −80 °C.

### 4.2. Bioinformatics Analysis

BLASTP (http://blast.ncbi.nlm.nih.gov, accessed on 10 March 2021) was used to search for homologous proteins in the *A. citrulli* genomes using amino acid sequences of HipA and HipB in *E. coli*. Gene sequences and amino acid sequences were obtained from KEGG (http://www.kegg.jp, accessed on 20 March 2021). To analyze the evolutionary relationships of *Aave_4519* and *Aave_4520* in *Acidovorax citrulli* AAC00-1 with homologous sequences from other bacterial species, we performed multiple sequence alignment using ClustalW in MEGA 7. Phylogenetic trees were constructed using the neighbor-joining method, with evolutionary distances calculated using the JTT-matrix-based model in MEGA 7. Bootstrap values were calculated using 1000 replicates to assess branch confidence. These details have been incorporated into the revised manuscript to enhance reproducibility. The interaction prediction and analysis were performed using STRING (http://version10.string-db.org, accessed on 13 April 2021). The amino acid conserved domains were compared using DNAMAN (version 8.0.8.789, Lynnon Biosoft, Quebec, QC, Canada).

### 4.3. Co-Transcription Test

Total RNA from the wild-type Aac5 strain was extracted using the bacterial RNA extraction kit (Yisheng, Shanghai, China), according to the manufacturer’s instruction, and the purity of the RNA was verified by agarose gel electrophoresis. The RNA was then reverse-transcribed into cDNA using a FastQuent RT Kit (TianGen, Beijing, China). Three pairs of primers, GZ4519F/R, GZ4520F/R, and gz1920F/R, were designed for *hipA*, *hipB*, and *hipA* + *hipB* gene sequences, respectively, using Primer 5 (the primer sequences can be found in Table A2). The gene sequences were based on the reference genome of *A. citrulli* AAC00-1 (Genbank number: CP000512.1). Aac5 genomic DNA and cDNA were used as templates for PCR amplification with the co-transcription primers *hipB* F 1, *hipB* R 1, *hipA* F 2, and *hipA* R 2. The co-transcription was confirmed using agarose gel electrophoresis.

### 4.4. Effect of Heterologous Expression of Predictable Proteins HipA on E. coli Growth

The primer pairs *hipA*-F/*hipA*-R and *hipB*-F/*hipB*-R were designed based on the sequence of the *hipA* gene (*Aave_4520*) and *hipB* gene (*Aave_4519*) in the AAC00-1 genome (Genbank accession number: CP000512.1). The *hipA* (1323 bp) and *hipB* (507 bp) genes were amplified from the genomic DNA of Aac5 using KOD-Plus-Neo (TOYOBO, Osaka, Japan) and the primers *hipA*-F/*hipA*-R and *hipB*-F/*hipB*-R. The ClonExpress II one-step cloning kit (Vazyme, Nanjing, China) was used to ligate *hipA* and *hipB* with vector pET-28a to obtain recombinant vectors *hipA*-pET-28a and *hipB*-pET-28a. These vectors were then transformed into competent *E. coli* BL21 and grown at 37 °C for 18 h. The empty plasmid, pET-28a, was transformed into *E. coli* BL21 as the control group. Single colonies were grown in LB + Kan liquid medium, with constant shaking (225 rpm) at 37 °C for 12 h. Gene-specific primers *hipA*-F/*hipA*-R and *hipB*-F/*hipB*-R were used for PCR verification, and the PCR products were sequenced at Huada Gene Co., Ltd. (Beijing, China) to confirm their accuracy. The constructed strains were stored at −80 °C with 50% glycerol in a 1:1 (*v*/*v*) ratio. Strains BL21-pET-28a, BL21-*hipA*-pET-28a, and BL21-*hipB*-pET-28a were grown on LB + Kan agar and incubated at 37 °C for 19 h. Single colonies were then grown in LB + Kan broth, with continuous shaking at 37 °C (225 rpm) for 12 h. The bacterial suspension was adjusted to a concentration of 3 × 10^6^ CFU/mL using a spectrophotometer (Biochrom, London, UK) and added to a cell culture plate at 200 μL per well. The plate was placed in a growth curve instrument (Bioscreen C PRO, Turku, Finland) to determine the growth capacity. The OD_600_ value was measured every 2 h for 72 h. Each sample was tested in ten replicates, and each experiment was independently conducted three times.

### 4.5. Bacterial Two-Hybrid Experiment

The interaction between HipA and HipB in Aac5 was investigated using a bacterial two-hybrid experiment. The ClonExpress II one-step cloning kit was used to ligate genes *hipA* and *hipB* with vectors pKT25m and pUT18Cm, respectively [29]. The resulting recombinant plasmids pKT25m-*hipA* and pUT18Cm-*hipB*, pUT18Cm-*hipA* and pKT25m-*hipB*, pUT18Cm-*hipA* and pKT25m-zip, pUT18Cm-*hipB* and pKT25m-zip, and pUT18Cm-*hipB* and pKT25m-zip were transformed into *E. coli* BTH101 in pairs to serve as the experimental groups. The plasmids pKT25m and pUT18Cm were transformed into *E. coli* BTH101 as a negative control. The plasmids pKT25m-zip and pUT18Cm-zip were totransformed into *E. coli* BTH101 as a positive control. Bacterial suspensions of the above strains with a volume of 10 μL were applied to LB + Kan + Amp agar containing X-gal and IPTG (0.5 mM) and incubated at 37 °C for 48 h.

### 4.6. Induced Expression and Purification of Target Proteins

The HipB protein with an N-terminal hexa histidine-tag (6*His tag) was expressed in *E. coli* BL21 using the pET-28a plasmid. Expression was induced when the *E. coli* BL21 bacterial suspension reached an OD_600_ value of 0.5. *E. coli* BL21 expressing HipB was induced with 1 mM IPTG and incubated for 14 h. Following centrifugation at 12,000 rpm and removal of the supernatant, the resulting pellet was resuspended in 40 mL of ice-cold 10 mM phosphate buffered saline for 2 min.

After cell lysis via sonication, the resulting lysate was subjected to centrifugation at 12,000 rpm for 20 min under refrigerated conditions (4 °C). The supernatant was loaded onto HisPurTMNi-NTA resin (TransGen Biotech, Beijing, China). A total 5–7 mL of Ni NTA resin packing was added to the chromatographic column. The soluble protein supernatant was mixed with the equilibrated filler, and the column was then washed once using 30 mM imidazole. The soluble protein supernatant was mixed with the protein filler using a silent mixer (Eppendorff, Hamburg, Germany) at 4 °C for 2 h. The mixture was then filled into the chromatographic column and eluted 3–5 times with imidazole eluent with a final concentration of 30 mM. The target proteins were collected using 20 mL of 30 mM imidazole eluting solution. Centrifugal concentration of the collected protein solution was performed using a 10 kDa Amicon Ultra filter (Merck, Shanghai, China).

### 4.7. Western Blot

The purified protein was detected using SDS-PAGE protein gel electrophoresis. The completed electrophoresis gel was carefully cut to obtain a small gel block containing the target protein. A suitable Polyvinylidene Fluoride (PVDF) membrane was selected according to the target protein and was immersed in a methanol solution for 15 s. The filter paper was soaked in the membrane buffer for 12 min and placed in the membrane analyzer in the order of filter paper, membrane, adhesive, and filter paper, at a voltage of 25 V for 10 min. The membranes were placed in TBST containing 5% skim milk and incubated at room temperature on a low-speed shaker (at 50 rpm) (ZHICHENG, Shang hai, China) for 90 min. Then, the membrane was placed in Anti-His Tag antibodies (Medical & Biological Laboratories Co., Ltd., Tokyo, Japan), diluted in a 1:5000 ratio, and incubated at room temperature for 1 h and 30 min. The PVDF membrane was soaked in Phosphate Buffered Saline with Tween (PBST) buffer and gently shaken on a small shaker. The luminescent agent and stabilizer were mixed in a 1:1 volume-to-volume ratio using a Western blot chemiluminescence detection kit. The mixture was then dropped onto the film. After a 5 min treatment in the dark, the film was placed into a luminescent imaging analyzer (Amersham, New York, NY, USA) for color imaging and the results were analyzed. The experiment was independently replicated three times.

### 4.8. EMSA

The EMSA experiment was conducted following the experimental method of Wang et al. [29]. The probe sequences were designed based on the *hipB* promoter region. Specifically, primers P_hipBA_-FAM-F/R and P_hipBA_-F/R were used to amplify fluorescently labeled and non-labeled *hipB* promoter fragments, respectively. The predicted promoter nucleic acid probes (P*_hipBA_* and P_hipBA_-FAM) were amplified by PCR and recovered. The PCR products were purified from agarose gels using a gel extraction kit, following the manufacturer’s instructions (Axygen, Beijing, China). Different final concentrations of proteins (36 μM, 24 μM, and 12 μM), nucleic acid probes (P*_hipBA_*-FAM, 60 ng/μL, and P*_hipBA_*, 120 ng/μL), and reaction buffer were completed in a 20 μL binding reaction system. The reaction system used in this study is shown in Table A3. After incubation at room temperature for 30 min, nucleic acid indicators were added, and the samples were applied to a 6% SDS-free protein gel. After protein electrophoresis was completed, nucleic acid staining was conducted for 30 min, and the binding between the protein and the nucleic acid probe was observed using a chemiluminescence imaging system (Merck, Shanghai, China).

### 4.9. Quantitative Real-Time (qRT) PCR Analyses of hipA and hipB in mRNA Expression Levels in A. citrulli Aac5

For measurements of *hipA* and *hipB* mRNA levels under chloramphenicol stress, *A. citrulli* Aac5 was grown overnight in KB at 28 °C, with constant shaking (150 rpm). After overnight growth, the cultures were diluted 1:100 and grown up to an OD_600_ of 0.5. The cultures were treated with 6.25 μg/mL chloramphenicol. RNA was extracted from cells harvested at 0, 15, and 30 h post-treatment using a bacterial RNA extraction kit (Yeasen, Shanghai, China) and reverse-transcribed into cDNA with a FastQuent RT Kit (TianGen, Beijing, China). qRT-PCR was performed using the SuperReal PreMix Plus (SYBR Green) kit (Tiangen, Beijing, China). qRT-PCR detection was conducted using the Step One Plus™ real-time PCR system.

For measurements of *hipA* and *hipB* mRNA levels under pH stress, *A. citrulli* Aac5 was grown overnight in KB at 28 °C with constant shaking (150 rpm). After overnight growth, the cultures were diluted 1:100 and grown to an OD_600_ of 0.5. Two milliliters of the treated culture was centrifuged at 13,000 rpm. The bacteria were cultivated using KB broth with pH values of 4.5, 5, and 7. At various times after stress induction (0, 15, and 30 h), the total RNA was extracted using a bacterial total RNA extraction kit (Yeasen, Shanghai, China). Reverse transcription and qRT-PCR were performed as described above.

To analyze the mRNA expression levels of *hipA* and *hipB* in plants, *A. citrulli* Aac5 was grown on KB for 48 h, resuspended from plates in sterile distilled water, and adjusted to an OD_600_ of 0.5 using a spectrophotometer (Biochrom, London, UK). Cotyledon inoculation experiments were performed on 7-day-old watermelon seedlings, which were kept in a greenhouse at 26–28 °C for 6 days. At the desired time points (6 h and 1–6 dai), an area of 1 cm^2^ around the inoculation point was cut and used for RNA extraction. Three pooled cotyledon segments were used for each biological repeat. Homogenized and weighed tissue segments were subjected to RNA extraction using the TRIzol method, followed by reverse transcription into cDNA using a FastQuent RT Kit (TianGen, Beijing, China). Each sample contained 1 μg of cDNA in a 20 μL reaction mix. The qRT-PCR was performed using the Step One Plus™ Real-Time PCR System.

The reference gene used was *rpoB*. The data were processed using the 2^−ΔΔCt^ method, and bar graph analysis was conducted using Prism 9 software, with a significance threshold of *p* < 0.05. All experiments were repeated three times.

## Data Availability

The original contributions presented in this study are included in the article.

## Appendix A

**Table A1:** Strains and plasmids used in this study.

Strains or Plasmids	Description	Reference or Source
Strains		
*Acidovorax citrulli* Aac5	Wild-type, Amp^R^	(Yan et al. [8])
BL21(DE3)	F-ompT hsdSB(rB-mB-)galdcm(DE3)	Biomed Company, Beijing
BTH101	F⁻ cya-99 araD139 galE15 galK16 rpsL1 hsdR2 mcrA1 mcrB1	This study
Plasmids		
pET-28a	Prokaryotic expression vector, Kan^R^	Zhao Lab collection
*hipA*-pET-28a	Prokaryotic expression vector carrying *hipA* gene, Kan^R^	This study
DH5ɑ-pRK600	Helper strain in tri-parental mating, Cm^R^	Zhao Lab collection
*hipB*-pET-28a	pET-28a carrying *hipB* gene, Kan^R^	This study
His-*hipA*-pET-28a	pET-28a carrying *hipA* gene with a His tag, Kan^R^	This study
His-*hipB*-pET-28a	pET-28a carrying *hipB* gene with a His tag, Kan^R^	This study
pUT18Cm-*hipA*	pUT18 carrying *hipA* gene, Amp^R^	This study
pUT18Cm-*hipB*	pUT18 carrying *hipB* gene, Amp^R^	This study
pKT25m-*hipB*	pKT25 carrying *hipB* gene, Kan^R^	This study
pKT25m-*hipA*	pKT25 carrying *hipA* gene, Kan^R^	This study

Kan^R^, Amp^R^, and Cm^R^ represented resistance of kanamycin, ampicillin, and chloramphenicol, respectively.

**Table A2:** Primers used in this study.

Primers	Primer Sequence (5′ > 3′)	Product Length/bp
*hipA*-F	ATGGTGGCGCTCGACGTGA	1323
*hipA*-R	TCAAGGGAGGCTGGCG	
*hipB*-F	ATGGACTATCCCATTCACCTGA	507
*hipB*-R	TTACCAAGAGCCCTTCTTGGG	
GZ4519F	GCCAACTCCGTGAGCACC	428
GZ4519R	TGACATCGGTGTTCGGGT	
GZ4520F	GCGTGGATGAATGGCGAA	1100
GZ4520R	GGCCCGGATGGTGTGTAT	
gz1920F	CTGGTGGTGAGAGATGACGC	1328
gz1920R	CGTAGGGCCAGACGGAAA	
*hipB*-pKT25-F	AGGGACTCTAGAGGATCCATGGACTATCCCATTCACCTGA	507
*hipB*-pKT25-R	TCTTAGTTACTTAGGTACTTACCAAGAGCCCTTCTTGGG	
*hipA*-pKT25-F	AGGGACTCTAGAGGATCCATGGTGGCGCTCGACGTGA	1323
*hipA*-pKT25-R	TCTTAGTTACTTAGGTACTCAAGGGAGGCTGGCG2	
*hipB*-pUT18-F	GTCGACTCTAGAGGATCCATGGACTATCCCATTCACC	507
*hipA*-pUT18-R	GAATTCGAGCTCGGTACCGGGAGGCTGGCG	
rpoB-F	GCGACAGCGTGCTCAAAGTG	104
rpoB-R	GCCTTCGTTGGTGCGTTTCT	
P_hipBA_-FAM-F	TCACCACGCTCTACAGGGACTT	396
P_hipBA_-FAM-R	CATCTATATCGGTTCGTGCCAA	
P_hipBA_-F	TCACCACGCTCTACAGGGACTT	396
P_hipBA_-R	TCACCACGCTCTACAGGGACTT	

**Table A3:** Electrophoretic mobility shift assay reaction system.

	System 1	System 2	System 3	System 4
HipB (μL)	3	2	1	1
FAM + DNA (μL)	2.2	2.2	2.2	2.2
Buffer (μL)	2	2	2	2
Non-specific Competitor DNA (μL)	0	0	0	4.8
ddH_2_O (μL)	2.8	3.8	4.8	0
